# Identifying discrepancies between clinical practice and evidence-based guideline in recurrent pregnancy loss care, a tool for clinical guideline implementation

**DOI:** 10.1186/s12884-023-05869-y

**Published:** 2023-07-28

**Authors:** A. Youssef, E.E.L.O. Lashley, N. Vermeulen, M.L.P. van der Hoorn

**Affiliations:** 1 Leiden University Medical Centre, Albinusdreef 2, Leiden, 2333 ZA the Netherlands; 2 European Society of Human Reproduction and Embryology, Nijverheidslaan 3 (BXL 7 – Gebouw 1), Strombeek, Bever, B – 1853 Belgium

**Keywords:** Guidelines, Implementation, RPL, Survey

## Abstract

**Background:**

Practice variation in recurrent pregnancy loss (RPL) care is common. International guidelines vary in their recommendations for the management of RPL couples, which could lead to an increase of cross border reproductive care. Currently, the Dutch RPL guideline is being adapted from the European Society for Human Reproduction and Embryology (ESHRE) guideline. We aim to identify discrepancies between RPL guidelines and RPL practice. These discrepancies could be considered in the development of a new guideline and implementation strategies to promote adherence to new recommendations.

**Methods:**

A nationwide survey on the management of RPL patients was conducted across all 107 hospital-based obstetrics and gynaecology practices in the Netherlands. The survey was sent via the Dutch Society for Obstetricians and Gynaecologists to all affiliated clinicians. The questionnaire consisted of 36 questions divided in four sections: clinician’s demographics, RPL definition, investigations and therapy. The data were compared to the recommendations given by the Dutch national guideline and the most recent guideline of the ESHRE.

**Results:**

All hospital-based practices (100%; n = 107) filled in the online questionnaire. The majority of respondents defined RPL similarly, as two or more pregnancy losses (87.4%), not obligatory consecutive (93.1%). More than half of respondents routinely perform thrombophilia screening ( 58%), although not advised by the ESHRE, while thyroid function (57%), thyroid auto-immunity (27%) and β2-glycoprotein antibodies (42%) in the context of antiphospholipid syndrome (APS) are recommended but investigated less often. Regarding parental karyotyping, 20% of respondents stated they always perform parental karyotyping, without prior risk assessment. because of RPL. Treatment for hereditary thrombophilia was frequently (43.8% (n = 137)) prescribed although not recommended. And finally, a considerable part (12–16%) of respondents prescribe medication in case of unexplained RPL.

**Conclusion:**

While many clinicians perform investigations recommended by the ESHRE, there is a considerable variation of RPL practice in the Netherlands. We identified discrepancies between RPL guidelines and RPL practice, providing possibilities to focus on multifaceted implementation strategies, such as educational intervention, local consensus processes and auditing and feedback. This will improve the quality of care provided to RPL patients and may diminish the necessity felt by patients to turn to multiple opinions or cross border reproductive care.

**Supplementary Information:**

The online version contains supplementary material available at 10.1186/s12884-023-05869-y.

## Background

Recurrent Pregnancy Loss (RPL) is defined as the loss of two pregnancies before 24th week gestation [1]. Despite extensive investigations, RPL remains unexplained in more than half of couples [1]. This affects couples’ psychological health and their quality of life. Therefore, an important role is preserved for the managing physician, to support and guide these couples through the many investigations and treatments.

In previous studies we have shown that although national and international guidelines exist, protocols still vary [1–6]. This high level of practice variation might lead to patients seeking care in various (inter)national centers, and be offered more extensive investigations and treatments.

In the Netherlands, the RPL guideline was published in 2007 by the Dutch Society for Obstetricians and Gynaecologists (NVOG) [7]. In the meantime, new evidence has been published regarding definition, investigations and treatments. Therefore, the guideline is in need of revision, which is currently conducted based on the ESHRE guideline [1]. This ESHRE guideline is developed based on up-to-date evidence with a strict guideline development methodology [8]. To make sure that clinicians will adhere to recommended investigations and treatments, different strategies to implement evidence-based guidelines are suggested [9]. One of the suggested strategies is to audit the current performance of health care providers. In this study we therefore conducted a nationwide cross-sectional survey on current clinical management of RPL patients across all 107 obstetric- and gynecology practices in the Netherlands, and compared the results with the most recent evidence-based guideline developed by the ESHRE .

## Methods

In this cross-sectional survey study an online questionnaire (Castor EDC) was sent to all 107 obstetric- and gynecology practices in the Netherlands in November 2020, with the Leiden University Medical Center as primary research centre. Eight of these hospitals were university hospitals, 62 teaching and 37 non-teaching hospitals. The questionnaire consisted of 36 questions in total which were divided in four sections: clinician’s demographics, RPL definition, investigations and therapy (Appendix 1). The survey was conducted over a three months period until all practices had completed the survey at least once (November 2020 until January 2021). The survey was sent via the Dutch Society for Obstetricians and Gynaecologists (to which all obstetrics and gynaecology practices are adjoined) to all affiliated clinicians. This includes residents, fertility doctors and medical specialists; all respondents participated the same survey. We aimed to obtain at least one response from 75% of all hospitals. After a second invitation to hospitals that had not yet responded, lead clinicians were contacted by mail.

Data is presented as percentages of respondents that indicated the specific answer choice over the total of respondents that answered the question. Between parentheses the number of replies to the corresponding question is given. Data were compared to the Dutch national [7] and the ESHRE [1] recommendations, as currently the ESHRE guideline is being adapted for a new RPL guideline in the Netherlands. Furthermore, data of university hospitals were compared to non-university hospitals using the Chi-squared test, with statistical significance when p < 0.05.

## Results

### Respondent demographics

All hospital-based practices (100%; n = 107) filled in the online questionnaire. A total of 446 entries were registered in the online questionnaire database. Of all entries, 315 were returned with 100% completion and 45 questionnaires were returned with at least 50% completion. The participants were primarily gynaecologists (71.7%; n = 320/446) or obstetrics and gynecology residents (22.4%; n = 100/446), the remaining participants (5.8%; n = 26/446) were 24 fertility doctors, one nurse and one medicine student. Half of all questionnaires were returned from non-university teaching hospitals (50.7%; n = 226/446), 23.1% (n = 103/446) by non-teaching hospitals and 21.1% (n = 94/446) by university hospitals. In addition, 20 entries were returned from private clinics and three remained unknown.

### Definition

The majority of respondents defined RPL as two or more pregnancy losses (87.4%; n = 346/394), not necessarily consecutive (93.1%; n = 367/394). Ectopic pregnancies (14.8%; n = 58/393), pregnancy of unknown location (31.3%; n = 123/393) and molar pregnancies (12.2%; n = 48/393) were included in the definition of RPL by a minority of respondents and biochemical pregnancies were included in the definition by 45.0% (n = 177/393) of respondents. Both spontaneous and assisted reproductive technology (ART) pregnancies were counted in RPL obstetric history by 93.8% of the respondents (n = 366/390) (Table 1). The Dutch guideline defines RPL as two or more pregnancy losses before 20 weeks of gestation, excluding ectopic, molar and biochemical pregnancies. The ESHRE guideline includes biochemical pregnancies and pregnancies of unknown location in the definition, as well as ART pregnancies.

**Table 1:** RPL definition components as used by respondents (in %) with information on the Dutch and ESHRE guideline recommendations for each component

	Number of respondents	Respondents (%)	Dutch guideline	ESHRE guideline
Number 2 3 More than 3	346/396 47/396 3/396	87 12 1	√	√
Consecutiveness Consecutive Non-consecutive	26/394 367/394	7 93	√	√
Pregnancy type included IUG Extra-uterine Molar Biochemical PUL	383/393 58/393 48/393 177/393 123/393	98 15 12 45 31	√	√ √ √
Origin Spontaneous ART and spontaneous	24/390 366/390	6 94	*	√
Obstetric history and relationship All pregnancies Only current relationship	226/388 162/388	58 42	√ √	*

IUG, intra-uterine pregnancy; PUL, pregnancy of unknown location with spontaneous regression

√ Recommended

^*^ Not indicated

### Investigations

The results of the investigations considered in this questionnaire are listed in Table 2, including the recommendations of both the Dutch national guideline and ESHRE. Most respondents initiate investigations after two pregnancy losses (87.3%; n = 324/371), and start with obtaining information on general aspects such as body weight and length (93.3%; n = 348/373), and lifestyle (90.2%; n = 343/373).

**Table 2:** Investigations performed by respondents (in %) with recommendations of the Dutch and ESHRE guideline

Investigations	Number of respondents	Respondents (%)	Dutch guideline	ESHRE guideline
General Aspects BMI Lifestyle Blood pressure	348/373 343/373 93/373	93 92 25	Recommended Yes Yes *	Recommended Yes Yes *
Genetic factors Female karyotype^†^ Male karyotype^†^ Pregnancy tissue array	262/363 260/363 8/363	72 72 2	On indication On indication Not recommended	On indication On indication Explanatory purpose only
Antiphospholipid syndrome ACA IgG ACA IgM Lupus Anticoagulant Anti-β2-glycoprotein IgG Anti-β2-glycoprotein IgM	318/350 294/350 321/350 148/350 129/350	91 84 92 42 37	Recommended Yes Yes Yes * *	Recommended Yes Yes Yes Yes Yes
Endocrine factors TSH TPO antibodies T4 and/or T3 Progesterone LH/FSH Glucose HbA1c	195/345 92/345 82/345 6/345 7/345 51/345 29/345	57 27 24 2 2 15 8	Not recommended * Not recommended Not recommended Not recommended Not recommended Not recommended	Recommended Recommended Not routinely recommended Not recommended Not recommended Not recommended Not recommended
Uterine factors 2D ultrasound 3D ultrasound HSG Hysteroscopy SIS MRI	266/347 31/347 11/347 55/347 71/347 4/347	77 9 3 16 21 1	*	Recommended Preferred technique
Male investigations Sperm DNA fragmentation Semen analysis Lifestyle	10/331 4/331 199/331	3 1 60	*	Explanatory purpose * *
Ongoing pregnancy hCG Progesterone Pregnancy tissue karyotype Pregnancy tissue array Ultrasound HbA1c Glucose	7/333 5/333 7/333 5/333 270/333 2/333 5/333	2 2 2 2 81 1 2	* * Not recommended * Yes * *	*
Thrombophilia Antithrombin – III APC-resistance APTT Factor II mutation Factor V Leiden Factor VIII Factor X Protein C Protein S Fibrinogen INR Thrombin time Homocysteine Plasminogen	105/358 83/358 37/358 80/358 105/358 41/358 16/358 120/358 127/358 18/358 9/358 20/358 206/358 4/358	29 23 10 22 29 12 5 34 36 5 3 6 58 1	On indication Yes Yes * Yes Yes Yes * Yes Yes * * * Yes *	Not routinely recommended
Infections CMV Chlamydia Gonorrhea	9/342 17/342 12/342	3 5 4	*	Not recommended
Immunological factors NK-cell plasma level NK-cell level in endometrial biopsy HLA typing and sharing HLA antibodies ANA antibodies	2/342 1/342 2/342 2/342 13/342	1 0 1 1 4	*	Not recommended Not recommended Not recommended Not recommended Explanatory purpose

^*^ Not indicated

^†^ Number indicated regards percentage of respondents that perform karyotyping according to individual risk assessment table

BMI: Body Mass Index; ACA: Anticardiolipin Antibodies; TSH: Thyroid Stimulating Hormone; TPO: Thyroid Peroxidase; HSG: Hysterosalpingography; SIS: Saline Infusion Sonohysterography; HbA1c: glycated hemoglobin; CMV: Cytomegalovirus; NK: Natural Killer; HLA: Human Leukocyte Antibiodies; ANA: Antinuclear Antibodies

According to the questionnaire, approximately 70% of respondents initiate parental karyotyping after risk assessment based upon the maternal age at second miscarriage, number of preceding miscarriages and history of miscarriages in either the siblings or in the parents [10]. 20% responded that they always perform parental karyotyping. Genetic testing on pregnancy tissue after miscarriages is performed by 2.2% (n = 8/363) of respondents. Both guidelines recommend karyotyping only on indication. The ESHRE recommends pregnancy tissue testing only for explanatory purposes.

Almost all participants offer APS investigations (98.9%; n = 346/350); lupus anticoagulant and anticardiolipin antibodies (ACA) are generally performed by most participants (see Table 2), anti-β2-glycoprotein antibodies testing is performed by less than half of respondents (IgM testing: 36.9%; n = 129/350 and IgG testing: 42.3%; n = 148/350). Both guidelines recommend APS testing.

Approximately half of the participants perform Thyroid Stimulating Hormone (TSH) testing (56.5%; n = 195/345), and 26.7% performs Thyroid Peroxidase (TPO) antibodies testing (n = 92/345). 24% of the participants indicate that they do not perform any endocrine testing (n = 107/345). The Dutch guideline does not recommend thyroid screening, while the ESHRE does recommend both function testing and auto-immunity thyroid testing.

Two-dimensional ultrasound was the most performed investigations according to this questionnaire (76.7%; n = 266/374). Three-dimensional ultrasound was preferred only by 8.9% of participants (n = 31/374). Three-dimensional ultrasound is the preferred technique as mentioned by the ESHRE guideline. The Dutch guideline does not mention testing for uterine malformations.

Regarding the male partner, respondents usually acquired male lifestyle information (60.1%; n = 199/331). A minority of responders also performed investigations, such as sperm DNA fragmentation (3.0%; n = 10/331) or semen analysis (1.2%; n = 4/331). This investigation is recommended by the ESHRE only for explanatory purposes. The Dutch guideline does not mention testing for male factors.

**Fig. 1:**
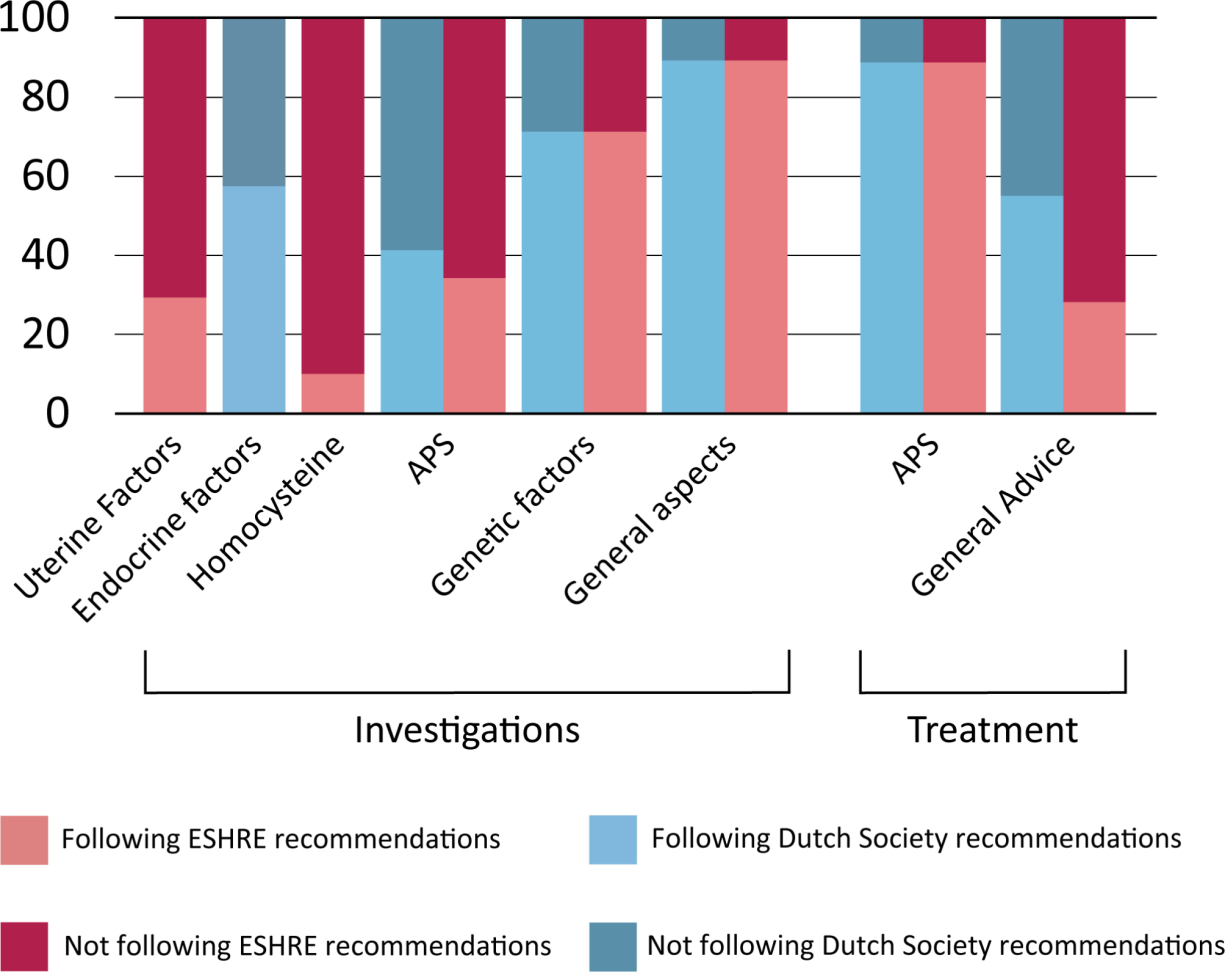
Representation of the percentage of respondents (Y-axis) that perform RPL care in line with the recommendations of the Dutch Society for Obstetricians and Gynaecologists and the ESHRE for investigations (left part on the X-axis) and treatment (right part on the X-axis). Categories on the X-axis contain all investigations and treatments that are recommended to be performed (non-recommended investigations and treatments are not included in percentages portrayed). General aspects include BMI, lifestyle and blood pressure. General advice refers to advice for smoking cessation, alcohol cessation, weight loss, and folic acid and vitamin D supplementation

An overview of the adherence to recommended investigations of the ESHRE and Dutch national guideline is provided in Fig. 1.

### Non-recommended investigations

The ESHRE and the Dutch guideline suggests not to screen for hereditary thrombophilia and/or hyperhomocysteinemia, unless in the context of research or in the presence of additional risk factors. Up to 58% (n = 206/358) of respondents indicated that they perform some form of thrombophilia investigations, and more than a quarter (29.1%; n = 104/358) indicated that they only perform hereditary thrombophilia investigations in case of additional risk factors.

### Treatment

The ESHRE advises to discuss health behaviour modifications such as cessation of smoking, striving for a normal range BMI, and limiting alcohol consumption. Smoking cessation was the most given general advice (95.4%; n = 310/325), followed by folic acid supplementation (89.2%; n = 290/325) and weight loss (78.2%; n = 254/325).

Respondents usually treated APS (59.7%; n = 190/318) in a next pregnancy, or referred patients for treatment to an internal medicine specialist or haematologist (28.9%; n = 92/318). The treatment consisted of Low Molecular Weight Heparin (LMWH) and aspirin, the order and starting time of these medications differed between respondents (such as starting from conception or from fetal heartbeat on ultrasound). The ESHRE recommends in treating patients diagnosed with APS in a next pregnancy with aspirin preconceptionally and LMWH in prophylactic dose starting at the day of a positive pregnancy test.

Treatment of hereditary thrombophilia was given by 43.8% (n = 137/313) of respondents and usually consisted of LMWH and/or aspirin. A third of respondents (35.5%; n = 111/313) referred patients for such treatment. Uterine septum correction was performed by 12.2% of respondents (n = 37/304). Patients with TPO antibodies were treated by half of respondents (51.5%; n = 158/307). Thyroid function was followed up during next pregnancy by 43.0% of the respondents (n = 132/307). The ESHRE does not recommend treatment for patients with hereditary thrombophilia or uterine septum, and states there is insufficient evidence to treat euthyroid women with thyroid antibodies.

When asked whether any treatments were given to unexplained RPL couples, 16.8% (n = 51/303) provided progesterone supplementation and 12.5% (n = 38/303) provided aspirin treatment in next pregnancy. A small percentage of respondents also prescribed other experimental treatments for patients with unexplained RPL (Table 3).

**Table 3:** Treatments performed by respondents (in%) with recommendations of the Dutch and ESHRE guideline

	Number of respondents	Respondents (%)	Dutch guideline	ESHRE guideline
General advice Smoking cessation Alcohol cessation Weight loss Folic acid Vitamin D	310/325 217/325 254/325 290/325 142/325	95 67 78 89 44	Recommended Yes Yes Yes * No	Recommended Yes Yes Yes Yes^+^ Yes^+^
Genetic factors Genetic counselling PGT	184/318 76/318	58 24	Recommended	Recommended
Uterine factors Septum resection	37/304	12	Not recommended	Not recommended
Endocrine antibodies (TPO)	26/307	9	Not recommended	Not recommended
Antiphospholipid syndrome	190/318	60	Recommended	Recommended
Thrombophilia	137/313	44	Not recommended	Not recommended
Unexplained RPL Progesteron hMG IVF HCG Thyroxine Corticosteroids IVIG Intralipids LMWH Aspirin Donor insemination Oocyte donation Endometrium scratching Cross border care referral	51/303 1/303 2/303 3/303 4/303 3/303 0/303 0/303 12/303 38/303 0/303 0/303 1/303 23/303	17 0 1 1 1 1 0 0 4 13 0 0 0 8	Not recommended	Not recommended

^*^ Not indicated

^+^ Not recommended in the context of improving live birth in RPL couples, but for general health purposes

An overview of the adherence to recommended treatments of the ESHRE and Dutch guideline is given in Fig. 1.

### ESHRE guideline

Half of the respondents had knowledge of the existence of the ESHRE guideline (49.3%; n = 149/302), and 38.4% (n = 116/302) indicated that they have implemented this guideline. University hospitals were more familiar with the ESHRE guideline (61.3% (n = 38/62) vs. 46.3% (n = 111/240); p = 0.035) and used this in daily practice (53.2% (n = 33/62) vs. 34.6% (n = 83/240); p = 0.007). Although a minority already uses the ESHRE guideline, three-quarter of respondents indicated their approval of the Dutch society of gynecology and obstetrics adapting the ESHRE guideline in Dutch practice (74.1%; n = 223/301).

### University versus non-university hospitals

Comparison between university and non-university hospitals showed a statistically significant difference in two questions regarding definition, namely including biochemical pregnancies (57.0% (n = 49/86) vs. 41.7% (n = 128/307); p = 0.012) and the inclusion of couple specific pregnancy losses (27.4% (n = 23/84) vs. 45.7% (n = 139/304); p = 0.003).

Anti-β2-glycoprotein IgG and IgM were investigated more often in university hospitals compared to non-university hospitals (IgG 56.2% (n = 41/73) vs. 38.6% (n = 107/277) and IgM 49.3% (n = 36/73) vs. 33.6% (n = 93/277) (p = 0.007 and p = 0.013).

Both TSH and TPO-antibodies investigations were more often performed in university hospitals (TSH 69% (n = 49/71) vs. 53.3% (n = 146/274); p = 0.017 and TPO-antibodies 36.6% (n = 26/71) vs. 24.1% (n = 65/274); p = 0.033). University hospitals also made more often use of 3D ultrasound for the investigation of uterine anomalies (31.9% (n = 23/72) vs. 2.9% (n = 8/275); p < 0.001).

Homocysteine screening was performed almost twice as often in non-university hospitals (35.2% (n = 25/71) vs. 63.1% (n = 181/287); p < 0.001). Furthermore, thrombophilia screening in the context of scientific studies was performed more often in university hospitals (16.9% (n = 12/71) vs. 3.8% (n = 11/287); p < 0.001).

## Discussion

In this cross-sectional survey study we audited the performance of healthcare providers on RPL guideline adherence. We observed that Dutch clinicians generally adhere to advised investigations and interventions (Fig. 1), though there is room for improvement.

In defining RPL, the ESHRE includes biochemical- and resolved pregnancies of unknown location. In our survey, < 50% of respondents followed this definition (Table 1), This may lead to an underestimation of RPL and exclusion of patients for further examination and treatment [11].

Considering discrepancies in investigations, we showed that 58% of respondents routinely perform thrombophilia screening, though not advised by the ESHRE [1]. The wide application of this screening can be explained by the long inclusion period of the ALIFE-2 study [12]. In the presence of a thrombophilic factor, clinicians may be tempted to start treatment, explaining the proportion of clinicians indicating treatment of hereditary thrombophilia in RPL couples (Table 3). Regarding parental karyotyping, 20% of respondents stated they always perform parental karyotyping, regardless of the risk assessment. Both the Dutch- as the ESHRE guideline recommends risk assessment prior to parental karyotyping, though this risk assessment is different amongst the guidelines, resulting in different number of karyotype testing. Regarding treatment discrepancies, we showed that a substantial portion of respondents advised progesterone or aspirin in patients with unexplained RPL. The ESHRE does not recommend treatment for patients with unexplained RPL, as no significant benefit was shown [13, 14]. A recent trial however showed a possible effect for the use of progesterone in women with ≥ 3 pregnancy losses presenting with early bleeding in a next pregnancy [15]. This could have implications for future recommendations on progesterone administration in patients with RPL.

To update the national RPL guideline and increase adherence to evidence-based RPL practice, currently adaptation of the ESHRE guideline is conducted in the Netherlands [1, 7]). While the ESHRE as the Dutch guidelines are similar in some respects, they also contain significant different recommendations either based on data published after finalization of the Dutch guideline, or based on differences in expert opinions in areas with a lack of studies. Implementation of the ESHRE guideline could therefore be hampered. Barriers identified in a European questionnaire [16] were the lack of a Dutch translation and the fact that guidelines are long and difficult to understand. Information on clinical practice with regards to these aspects is helpful to identify discrepancies for better implementation of future evidence-based guidelines.

Recently, Manning et al. have performed a comparable study in the UK and showed equivalent results regarding practice variation [17]. They explained that in many practices dedicated RPL specialists were absent, who can strive for a consistent management of RPL couples. Our results support a similar conclusion based on practice variation between university and non-university clinics. Indeed, university hospitals show more often a definition and policy congruent with the current ESHRE guideline.

We believe that a multifaceted implementation strategy could help improving guideline adherence, and thus evidence-based practice and also reduce unnecessary medical costs [17]. This strategy implies educational intervention, such as disseminating of summary of the recommendations or the development of a web-based tool. In addition, local consensus processes for care that lack scientific evidence could help minimizing practice variation. And finally, auditing of healthcare workers’ performance and feedback, as we performed in this study, could act as an incentive to improve a clinician’s management of RPL patients. Multifaceted implementation strategies are however not widely present regarding guideline implementation.

A major strength of our study is that we achieved 100% response rate, as all hospital-based practices have participated in this survey. This resulted in the elimination of sampling bias. Our study confirms previous findings of variation in practice and limited adherence to national guidelines [2, 6, 18, 19]. We showed that ESHRE is rightfully concerned about the implementation of the RPL guideline [20].

A limitation of this study is that it was not possible to elucidate why clinicians persist or refrain from certain investigations and therapeutic options. This could have demonstrated a rationale for the demonstrated practice variation. Furthermore, survey studies are susceptible to desirability bias. It was not possible to measure whether participants gave any desirable answers, knowing guideline recommendations but performing otherwise in daily practice.

## Conclusion

While many clinicians perform investigations recommended by the ESHRE, we also identified considerable discrepancies. Clinicians tend to rely more on guidelines published by national societies. To limit practice variation and thereby delivering care up to maximum standards, it is necessary that efforts of both overarching societies such as the ESHRE and local societies are collaborating in implementation of up-to-date guidelines. Using implementation strategies to improve guideline adherence will ultimately lead to better care delivered to RPL patients.

## Electronic supplementary material

Below is the link to the electronic supplementary material.

Supplementary Material 1

## Data Availability

The datasets generated and analysed during the current study are not publicly available but are available from the corresponding author on reasonable request.

## List of abbreviations

ACA: Anticardiolipin antibodies
ANA: Antinuclear antibodies
APS: Antiphospholipid syndrome
ART: Artificial reproductive therapy
CRBC: Cross-border reproductive care
ESHRE: European society for human reproduction and embryology
HLA: Human leukocyte antigen
LMWH: Low molecular weight heparin
NVOG: Nederlandse vereniging voor obstetrie en gynaecologie (Dutch Society for Obstetricians and Gynaecologists)
RPL: Recurrent pregnancy loss
TPO: Thyroid peroxidase
VTE: Venous thromboembolism
