# Supplementary material for: Identifying discrepancies between clinical practice and evidence-based guideline in recurrent pregnancy loss care, a tool for clinical guideline implementation
Source: BMC Pregnancy Childbirth. 2023 Jul 28;23:544. doi: 10.1186/s12884-023-05869-y (PMC10386208; doi:10.1186/s12884-023-05869-y)
Supplement: Supplementary file 1 — Supplementary Material 1 [file 12884_2023_5869_MOESM1_ESM.docx]

Appendix I – Online questionnaire

Demographics

1. What is your profession?
   - Gynaecologist
   - Fertility doctor
   - Resident
   - Intern
   - Midwife
   - Other
2. What type of hospital do you work in?
   - University hospital
   - Non-university teaching hospital
   - Non-university non-teaching hospital
   - Private clinic
3. What is the name of the hospital you work in (open question)?

RPL definition

1. What aspects do you use in the RPL definition?
   - Number of pregnancy losses
   - Consecutive/non-consecutive pregnancy losses
   - Primary vs secondary RPL
   - Pregnancy location (EUG, PUL, intra-uterine)
   - Gestational age of pregnancy
2. I define RPL as
   - 2 pregnancy losses
   - 3 pregnancy losses
   - More than 3 pregnancy losses
   - I don’t know
3. I include the following pregnancy losses in the RPL definition
   - Consecutive pregnancy losses
   - Both consecutive and non-consecutive pregnancy losses
   - I don’t know
4. I include the following pregnancies in the RPL definition
   - Intra-uterine pregnancy
   - Extra-uterine pregnancy
   - Pregnancy of unknown location with spontaneous regression
   - Molar pregnancy
   - Biochemical pregnancy
5. Do you only include spontaneous pregnancies in the RPL definition?
   - Yes
   - No, also artificial reproductive technology (ART) assisted pregnancies
6. Do you only include pregnancy losses from current couple relationship in the RPL definition?
   - Yes
   - No
7. What is your preference for RPL definition (open question)?

Investigations

1. Do you offer investigations to RPL coupels?
   - Yes
   - No
2. When do you offer investigations?
   - To all referred couples
   - To couples with 2 pregnancy losses
   - To couples with 3 pregnancy losses
3. What general aspects do you investigate?
   - Weight and length
   - Lifestyle
   - Blood pressure
   - I don’t offer general apsects investigation
   - Other (with open textbox)
4. What genetic testing do you offer?
   - Always male karyotyping
   - Always female karyotyping
   - Male karyotyping according to risk
   - Female karyotyping according to risk
   - Pregnancy tissue karyotyping
   - I never offer karyotyping
   - Other (with open textbox)
5. What hereditary thrombophilia testing do you offer?
   - Antithrombin – III
   - APC-resistance
   - APTT
   - Factor II mutation
   - Factor V Leiden
   - Factor VIII
   - Factor X
   - Protein C
   - Protein S
   - Fibrinogen
   - INR
   - Thrombin time
   - Homocysteine
   - Plasminogen
   - Only in the context of scientific research
   - Only in the presence of risk factors
   - I don’t offer hereditary thrombophilia testing
   - Other (with open textbox)
6. What antiphospholipid syndrome testing do you offer?
   - Anticardiolipin antibodies IgG
   - Anticardiolipin antibodies IgM
   - Lupus Anticoagulant
   - Anti-β2-glycoprotein IgG
   - Anti-β2-glycoprotein IgM
   - Thrombocyte count
   - Antinuclear antibodies
   - I don’t offer antiphospholipid syndrome testing
   - Other (with open text box)
7. What endocrine testing do you offer?
   - Thyroid Stimulating Hormone (TSH)
   - Thyroid peroxidase (TPO) antibodies
   - T4 and/or T3
   - Progesterone
   - LH/FSH
   - Glucose
   - HbA1c
   - I don’t offer endocrine testing
   - Other (with open textbox)
8. What uterine anomalies testing do you offer?
   - 2D ultrasound
   - 3D ultrasound
   - Hysterosalpingography
   - Hysteroscopy
   - Saline infusion sonohysterography
   - MRI
   - I never offer uterine anomalies testing
   - Other (with open textbox)
9. What infectious diseases testing do you offer?
   - Cytomegalovirus
   - Chlamydia
   - Gonorrhea
   - I never offer infectious diseases testing
   - Other (with open textbox)
10. What immunological testing do you offer?
    - NK-cell plasma level
    - NK-cell level in endometrial biopsy
    - Human Leukocyte Antibodies (HLA) typing and sharing
    - HLA antibodies
    - Antinuclear antibodies
    - I don’t offer immunological testing
    - Other (with open textbox)
11. Do you offer any other investigations not previously mentioned?
    - Yes (with open textbox)
    - No
12. What investigations do you offer when a RPL couples gets pregnant?
    - hCG
    - Progesterone
    - Pregnancy tissue karyotype (after pregnancy loss)
    - Pregnancy tissue array (after pregnancy loss)
    - Ultrasound
    - HbA1c
    - Glucose
    - I don’t offer investigations when RPL couples get pregnant
    - Other (with open textbox)
13. Do you maintain an age limit when offering investigations? If so, what age?
    - Yes (with open textbox)
    - Yes, but only in case of karyotyping (with open textbox)
    - No
14. What male investigations do you offer?
    - Sperm DNA fragmentation
    - Semen analysis
    - Lifestyle
    - I don’t offer male investigations
    - Other (with open textbox)

Advice and Treatment

1. What general advices do you offer?
   - Smoking cessation
   - Referral to smoking cessation program
   - Alcohol cessation
   - Weight loss
   - Referral to dietician
   - Folic acid
   - Discourage caffeine intake
   - Vitamin D
   - I don’t offer general advices
   - Other (with open textbox)
2. Do you offer supportive care? If so, what does supportive care consist of?
   - Yes (with open textbox)
   - No
3. Do you treat RPL patients with antiphospholipid syndrome? And if so, what does treatment regimen consist of (drugs, doses, starting point)?
   - Yes
   - No
   - No but I refer patients to another specialist
4. Do you treat RPL patients with hereditary thrombophilia? And if so, what does treatment regimen consist of?
   - Yes
   - No
   - No but I refer patients to another specialist
5. Do you treat euthyroid RPL patients with TPO antibodies? And if so, what does treatment regimen consist of?
   - Yes
   - No
   - No but I recommend thyroid function follow-up
6. How do you deal with RPL patients with chromosomal translocations (Open textbox)?
7. How do you treat RPL patients with uterine anomalies?
   - Septum resection
   - Myomectomy
   - Cerclage
   - I don’t treat patients with uterine anomalies
   - Other (with open textbox)
8. Do you offer any of the following investigations to couples with unexplained RPL?
   - Progesteron
   - hMG
   - IVF
   - HCG
   - Thyroxine
   - Corticosteroids
   - IVIG
   - Intralipids
   - LMWH
   - Aspirin
   - Donor insemination
   - Oocyte donation
   - Endometrium scratching
   - I don’t offer any treatment to couples with unexplained RPL
   - Other (with open textbox)
9. Do you refer couples to cross border clinics? And if so, why do you refer them?
   - Yes (with open textbox)
   - No

ESHRE guideline

1. Do you know the 2017 ESHRE guideline on RPL?
   - Yes
   - No
2. Do you use the 2017 ESHRE guideline?
   - Yes
   - No
3. Do you believe that the Dutch Society for Obstetricians and Gynaecologists should adapt the 2017 ESHRE guideline?
   - Yes
   - No

Any other comments (open textbox)
